# Efficacy of umbilical cord mesenchymal stromal cells for COVID-19: A systematic review and meta-analysis

**DOI:** 10.3389/fimmu.2022.923286

**Published:** 2022-08-29

**Authors:** Cong-wen Yang, Ru-dong Chen, Qing-run Zhu, Shi-jie Han, Ming-jie Kuang

**Affiliations:** ^1^ Department of Neurosurgery, Weifang Medical University, Weifang, China; ^2^ Department of Orthopedics, Shandong Provincial Hospital Affiliated to Shandong First Medical University, Jinan, Shandong, China

**Keywords:** COVID-19, umbilical cord mesenchymal stromal cells (UC-MSCs), immunomodulation, adverse events and severe adverse events, the Mortality rate,

## Abstract

**Objectives:**

A major challenge for COVID-19 therapy is dysregulated immune response associated with the disease. Umbilical cord mesenchymal stromal cells (UC-MSCs) may be a promising candidate for COVID-19 treatment owing to their immunomodulatory and anti-inflammatory functions. Therefore, this study aimed to evaluate the effectiveness of UC-MSCs inpatients with COVID-19.

**Method:**

Medline, Embase, PubMed, Cochrane Library, and Web of Science databases were searched to collect clinical trials concerning UC-MSCs for the treatment of COVID-19. After literature screening, quality assessment, and data extraction, a systematic review and meta-analysis of the included study were performed.

**Results:**

This systematic review and meta-analysis were prospectively registered on PROSPERO, and the registration number is CRD42022304061. After screening, 10 studies involving 293 patients with COVID-19 were eventually included*. Our* meta-analysis results showed that UC-MSCs can reduce mortality (relative risk [RR] =0.60, 95% confidence interval [CI]: [0.38, 0.95], P=0.03) in COVID-19 patients. No significant correlation was observed between adverse events and UC-MSC treatment (RR=0.85, 95% CI: [0.65, 1.10], P=0.22; RR=1.00, 95%CI: [0.64, 1.58], P=1.00)*. In* addition, treatment with UC-MSCs was found to suppress inflammation and improve pulmonary symptoms.

**Conclusions:**

UC-MSCs hold promise as a safe and effective treatment for COVID-19.

**Systematic Review Registartion:**

PROSPERO, identifier CRD42022304061

## 1 Introduction

The COVID-19 pandemic was first reported at the end of 2019 and is caused by the novel severe acute respiratory syndrome coronavirus 2 (SARS-CoV-2) (1). This highly infectious virus has spread worldwide, leading to the pandemic (2). The World Health Organization has reported more than 50 million confirmed cases of COVID-19, including more than 6 million deaths, worldwide as of June 2022; 30%–40% mortality has been observed in critically ill patients with COVID-19 (3). Severe COVID-19 is most evident when it involves cytokine release syndrome (CRS), acute respiratory distress syndrome and multi-organ failure (4). The uncontrolled systemic inflammatory response is thought to be an essential factor in the deterioration and death of COVID-19 patients (5). The current treatment for systemic inflammation is high doses of corticosteroid injections; systemic corticosteroid use has been shown to reduce 28-day mortality in critically ill COVID-19 patients (6). However, heavy use of corticosteroids can lead to many serious sequelae, such as osteoporosis and recurrent infections (7). In addition, clinical trials have demonstrated the effectiveness of interleukin (IL)-6 receptor blockers (8), antiviral drugs (9) and monoclonal antibodies (10) in the treatment of COVID-19. However, the situation to fight against COVID-19 remains critical with mutations in the virus, and new therapeutic approaches should be explored.

Mesenchymal stromal cells (MSCs) have unique immunomodulatory and regenerative characteristics that may represent promising treatment agents for COVID-19 (11). MSC therapy may prevent the immune system from releasing a storm of cytokines and promote endogenous repair through the repair properties of the stem cells (12). Numerous studies have shown that the immunomodulatory mechanisms of MSCs play an important role in inflammation (13–16). In addition, after intravenous injection of MSCs, many cells accumulate in the pulmonary area, and their immunomodulatory effects protect the alveolar epithelial cells, restore the lung microenvironment, prevent lung fibrosis and treat lung dysfunction (12).

MSCs can be isolated from human umbilical cords, bone marrow, endometrium, menstrual blood, fat and other tissues (17). Human umbilical cord mesenchymal stromal cells (UC-MSCs) are derived from the umbilical cord after delivery and are typical adult stem cells (18). Their advantages over other MSCs sources include low immunogenicity, non-invasive harvesting procedures, ease of *in vitro* expansion and ethical access (19). Evidence accumulated to date has shown that allogeneic UC-MSCs are safe for use in a variety of diseases (20). Currently, UC-MSCs are used to treat autoimmune diseases, promote haematopoiesis and repair tissues and organs. Therefore, we performed a systematic review and meta-analysis to evaluate the efficacy of UC-MSCs for COVID-19 treatment.

## 2 Materials and methods

### 2.1 Search strategy

This systematic review cum meta-analysis was prospectively registered on the International Prospective Register of Systematic Reviews (PROSPERO) (21) under the registration number CRD42022304061. The Preferred Reporting Items for Systematic Reviews and Meta-Analyses (PRISMA) guidelines (22) (**Additional File 1**) and Cochrane Handbook (23) were used to evaluate the quality of the results of all included studies to ensure that the results of our meta-analysis were reliable and authentic (24).

**Figure 1:**
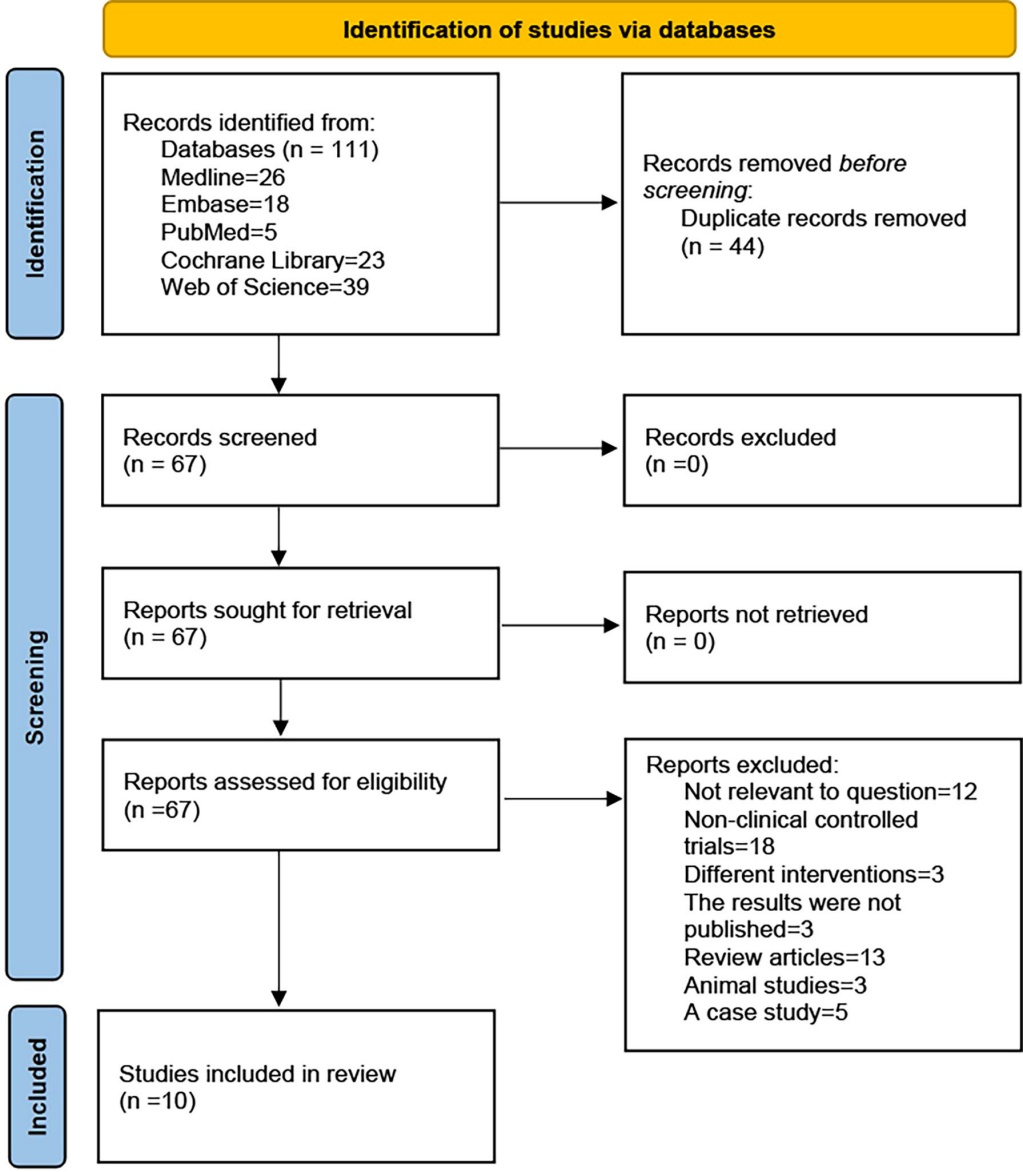
PRISMA 2020 flow diagram. A total of 111 records were retrieved, after inclusion and exclusion criteria, the final 10 studies were included.

A systematic review and meta-analyses were performed to identify relevant randomised controlled trials (RCTs) and non-RCTs using electronic databases, including the Medline, Embase, PubMed, Cochrane Library and Web of Science, up to December 2021. The search keywords used MeSH terms which were (COVID-19 or SARS-COV-2) AND (umbilical cord-derived mesenchymal stromal cells or mesenchymal cells from the umbilical cord). A flowchart of the literature screening process is presented in **Figure 1**.

### 2.2 Inclusion and exclusion criteria

#### 2.2.1 Participants/population

The inclusion criteria were (a) patients aged 18–95 years, (b) those who were critically ill with confirmed COVID-19 using real-time polymerase chain reaction and (c) those who provided signed informed consent.

The exclusion criteria included (a) patients presenting any history of malignancy, (b) those with pregnancy or a positive pregnancy test and (c) those who participating in another clinical trial within the past 3 months.

#### 2.2.2 Interventions and exposures

The inclusion criteria were (a) studies on UC-MSCs, (b) those wherefrom which UC-MSCs were possibly obtained from the autologous or allogeneic tissues and (c) those involving all routes of administration, such as intravenous, aerosol inhalation, and intramuscular approaches and (d) possible administration of other therapeutic agents (antivirals, anti-cytokine agents, etc.).

#### 2.2.3 Comparator(s)/control

Patients receiving conventional therapies for COVID-19 treatment (antivirals, immunomodulatory drugs, anti-cytokine drugs, etc.) and placebo will be included. No control group was set and studies comparing UC-MSCs therapy with other treatments were excluded.

#### 2.2.4 Types of study to be included

The inclusion criteria were (a) studies involving only published clinical trials including RCT and non-RCT cohort studies, (b) studies with a follow-up of at least 80% and at least one primary outcome and (c) those with complete treatment outcomes.

Review articles, animal studies, case studies that were not relevant to the question and data that were not extractable were excluded.

### 2.3 Outcomes

The primary outcomes included mortality rate, adverse events (AEs), and severe adverse events (SAEs). The secondary outcomes included supplemental oxygen, duration of oxygen therapy, hospital stay, pulmonary function, immune cells, inflammatory markers, pro-inflammatory cytokines, pulmonary imaging changes, pulmonary function and prognosis.

### 2.4 Data extraction

Data were extracted independently by two reviewers, and controversial data were discussed and agreed on. Eligible articles were analysed for data extraction to ensure the accuracy of the data. For analysis, we extracted data, including publication date, authors, study site, study design, interventions, sample size, follow-up time, sex, age, and outcomes. The authors of the corresponding RCTs were contacted, if required, to ensure the integration of information and obtain any missing data (25).

### 2.5 Risk of bias and quality assessment

The Modified Jadad scale, including random sequence production, allocation concealment, blinding method, and withdrawal, was used to assess the quality of RCTs, and studies scoring > 4 indicated high quality. The Cochrane recommends using Risk of Bias In Non-randomized Studies of Interventions (ROBINS-I) for risk of bias assessment of non-RCTs and observational studies of interventions (26).

### 2.6 Statistical analysis and the assessment of publication bias

The Review Manager software (version 5.3) was used to perform meta-analyses. Statistical heterogeneity was assessed using the I^2^. I^2^ values <30% were considered to have low heterogeneity and fixed effects models were used. When I^2^ >30%, >50%, and >75% were considered to indicate moderate, substantial, and considerable heterogeneity, a random effects model was used to analyze the data (27). For dichotomous outcomes, the results were presented as relative risk (RR) with a 95% confidence interval (CI) (28). The mean difference (MD) or standardised mean difference (SMD) was used to assess continuous outcomes, such as the duration of oxygen therapy and the length of stay with 95% CI. To reduce heterogeneity, subgroup analyses were performed.

Some studies have reported the median, first and third quartiles and maximum and minimum values. To perform a valid meta-analysis of continuous variables, these data were transformed into means and standard deviations using the Box-Cox transformation method (29).

Qualitative assessment of the funnel plot to determine publication bias, and visual inspection to determine whether there are any asymmetries. Making funnel plots with “STATA” software (version 14).

## 3 Results

### 3.1 Search results

A total of 111 records were obtained by searching the electronic database. After excluding duplicates, we found 67 records, of which 57 not meet the inclusion criteria were excluded (Including 12 not related to the research question, 18 were non-clinical controlled trials, 3 had unpublished results, 13 reviews, 3 animal studies, 5 case study, and 3 study interventions differed in that they used non-UC-MSCs). Finally, only 10 studies were included in this systematic review (**Figure 1**). The study included six RCTs (30–36), two non-RCTs (37, 38) and two prognostic analyses of RCTs (39, 40).

### 3.2 Study characteristics

Quality assessment of the six RCTs according to the Modified JADAD indicated that all were high quality. Non-RCTs and observational studies were conducted using ROBINS-I for risk of bias assessment. Meng et al., Lei et al. and Feng et al. was evaluated for low risk bias, and Wei et al. was evaluated for moderate risk bias (Scoring details in **Table 1**). UC-MSCs for CIVID-19 is a new therapy, so only a small number of studies were included. There was heterogeneity in study inclusion criteria: In Giacomo and Monsel’s study, COVID-19 patients had developed ARDS. There is no uniform standard of administration for stem cell therapy, thus leading to heterogeneity. And the majority of patients with complications, like diabetes and hypertension. **Table 2** summarises the characteristics of the included studies, and **Table 3** shows the baseline characteristics of patients included in this review.

**Table 1:** The Risk of Interventions (ROBINS-I) assessment.

References	Risk of bias judgement							
	Bias due to confounding	Bias in selection of participants into the study	Bias in classification of interventions	Bias due to deviations from intended interventions	Bias due to missing data	Bias in measurement of outcomes	Bias in selection of the reported result	Overall bias
Meng et al.	Low	Low	Low	Low	Moderate	Moderate	Low	Low
Wei et al.	Low	Low	Low	Moderate	Moderate	Moderate	Low	Moderate
Lei et al.	Low	Low	Low	Low	Moderate	Low	Low	Low
Feng et al.	Moderate	Low	Low	Low	Moderate	Low	Low	Low

**Table 2:** Characteristics of included studies.

References	Year	Country	CasesUC-MSCs/Control	AgeUC-MSCs/Control	Gender% male	Study Design	Quality score	Quality assessment
Lei Shi et al.	2021	China	65/35	60.72/59.94	56.92%/54.29%	RCT	7	modified Jadad scale
Dilogo et al.	2021	Indonesia	20/20	NM	NM	RCT	7	modified Jadad scale
Giacomo et al.	2021	USA	12/12	58.58/58.63	41.7%/66.7%	RCT	7	modified Jadad scale
Lei Shu et al.	2020	China	12/29	61/58	66.67%/55.17%	RCT	4	modified Jadad scale
Kouroupis et al.	2021	USA	12/12	58.58/58.63	41.7%/66.7%	RCT	7	modified Jadad scale
Monsel et al.	2022	France	21/24	64/63.2	81%/83.3%	RCT	6	modified Jadad scale
Meng et al.	2020	China	9/9	45.1/49.6	77.78%/44.45%	non-RCT	Low risk	ROBINS-I
Wei et al.	2021	China	12/13	67/68	58.3%/38.5%	non-RCT	Moderate risk	ROBINS-I
Lei et al.	2021	China	65/35	60.72/59.94	56.92%/54.29%	Prognosis	Low risk	ROBINS-I
Feng et al.	2021	China	8/20	50.5/51	50%/45%	Prognosis	Low risk	ROBINS-I

NM, not mentioned; RCT, randomized controlled trials; non-RCT, non-randomized controlled trials; ROBINS-I, Risk of Bias in Non-randomized Studies of Interventions.

**Table 3:** Baseline patient characteristics.

References	State of anillness	Interventions	Dose	Number of infusions	Comorbidities	Concomitant treatments	Observed duration
Lei Shi et al.	severe COVID-19	UC-MSCs/Placebo	4.0 × 10^7^cells for each	Three injections	Diabetes, Hypertension, Chronic bronchitis Chronic obstructive	Antiviral drugs, Antibiotics Corticosteroids	28d
Dilogo et al.	severe COVID-19	UC-MSCs/Placebo	1×10^6^/kg body weight	Single-injection	Diabetes, Hypertension, Tuberculosis Chronic kidney disease, Coronary, Arterial disease, Congestive heart failure,	NM	15d
Giacomo et al.	COVID-19 ARDS	UC-MSCs/Placebo	100 ± 20×10^6^UC-MSCs each	Two injections on day 0 and day 3	Diabetes, Hypertension, Obesity, Cancer, Heart disease	Heparin, Remdesivir, Corticosteroids Convalescent plasma, Tocilizumab, Alteplase, Hydroxychloroquine,	31d
Lei Shu et al.	severe COVID-19	UC-MSCs/ Conventional therapy	2 × 10^6^cells/kg.	Single-injection	Diabetes, Hypertension	Supplemental oxygen, Antiviral agents, Antibiotic agents Glucocorticoid therapy	14d
Monsel et al.	COVID-19 ARDS	UC-MSCs/Placebo	0.9 ± 0.1×10^6^ UC-MSCs/kg per dose	Three intravenous infusions of 106 UC-MSCs/kg	Chronic obstructive pulmonary disease, Atrial, fibrillation, Hypertension, Stroke Coronary artery disease	corticosteroid	28d
Kouroupis et al.	severe COVID-19	UC-MSCs/ Conventional therapy	3 × 10^7^cells each infusion	Three injections	Hypertension, Diabetes, Fatty liver, Disease, Asthma	Antivirals treatment Steroids treatment	14d
Meng et al.	COVID-19	UC-MSCs/ Conventional therapy	5.20-7.20×10^7^UC-MSCs	Single-injection	Diabetes, Hemorrhagic cerebral infarction	Antiviral therapy Methylprednisolone	14d

NM, not mentioned.

### 3.3 Primary Outcomes

#### 3.3.1 The mortality rate

**Figure 2:**
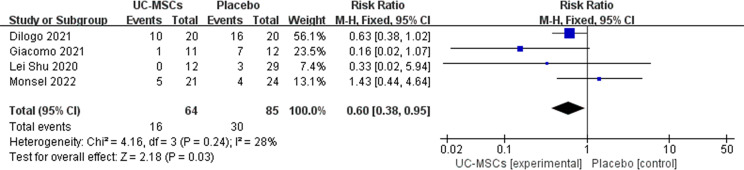
The effect of UC-MSCs therapy by forest plot diagram on COVID-19 mortality rate.

The mortality rates in six RCTs were analysed, and two of them showed no patient deaths in both control and experimental groups; therefore, a meta-analysis was performed with four of the remaining studies (**Table 4**). Heterogeneity was observed in the forest plots (I^2 =^ 28%, p=0.24). Low heterogeneity was noted; therefore, a fixed-effects model was used. RR with 95% CI were used to assess the results of the dichotomous method. The meta-analysis results showed a significant difference between the UC-MSC and control groups in terms of mortality rate (RR=0.60, 95% CI: [0.38, 0.95], P=0.03; **Figure 2**). This result indicated lower mortality in the UC-MSC group than that in the control group.

**Table 4:** Primary outcomes.

References	Mortality rate		AEs		SAEs	
	UC-MSCs	Control	UC-MSCs	Control	UC-MSCs	Control
Lei Shi et al.	0/65	0/35	The incidence of adverse events was 55.38%. The most common adverse event was an increase in lactic acid dehydrogenase (13.85%).	The incidence of adverse events was 60%. The most common adverse event was an increase in lactic acid dehydrogenase (20%).	One case experienced pneumothorax.	No serious adverse events occurred.
Dilogo et al.	10/20	16/20	No life-threatening complications or acute allergic reactions.			
Giacomo et al.	1/11	7/12	Number of AEs reported: 35/88 Number of subjects with AEs: 8/12	Number of AEs reported: 53/88 Number of subjects with AEs: 11/12	Number of SAEs reported: 2/18 Number of subjects with SAEs:2/10	Number of SAEs reported: 16/18 Number of subjects with SAEs: 8/10
Lei Shu et al.	0/12	3/29	No adverse reactions (such as rash, allergic reaction, and febrile reaction after infusion).			
Monsel et al.	5/21	4/24	Thirty-six (80%) patients suffered an adverse event by day 14. Eighteen (40%) suffered adverse events thereafter. Only one patient in the UC-MSC group suffered diarrhea thought to be possibly related to treatment.			
Meng et al.	0/9	0/9	Two patients developed transient facial flushing and fever immediately on infusion, which resolved spontaneously within 4h. Another patient had a transient fever (38°C) within 2 h.	NM	No serious adverse events.	NM
Wei et al.	1/12	0/13	No AEs or SAEs occurred.			

NM, not mentioned; AEs, Adverse Events; SAEs, Severe Adverse Events.

##### 3.3.1.1 AEs and SAEs

**Figure 3:**
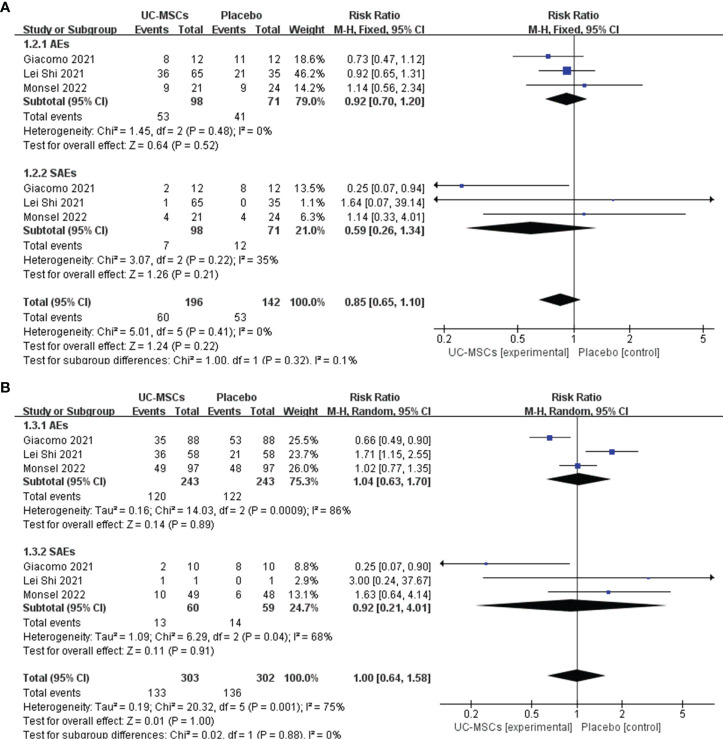
The effect of UC-MSCs therapy by forest plot diagram on AEs and SAEs. **(A)** The number of patients experiencing AEs and SAEs; **(B)** The number of types of AEs and SAEs.

We analysed the adverse effects of treatment with UC-MSCs in two aspects: the number of patients experiencing AEs in each group and the number of adverse effects that occurred in each group. Subgroup analysis was used to evaluate AEs and SAEs. The results of the meta-analysis are shown using forest plots. The number of patients experiencing AEs and SAEs in the two groups were compared (RR=0.85, 95% CI: [0.65, 1.10], P=0.22; AEs: RR=0.92, 95% Cl: [0.70, 1.20], p=0.52; SAEs: RR=0.59, 95% Cl: [0.26, 1.34], p=0.22, **Figure 3A**). The number of types of AEs and SAEs in the two groups were compared (RR=1.00, 95% CI: [0.64, 1.58], P=1.00; AEs: RR=1.04, 95% Cl: [0.63, 1.70], p=0.89; SAEs: RR=0.92, 95% Cl: [0.21, 4.01], p=0.91, **Figure 3B**). Both meta-analyses showed no significant difference between the UC-MSC and control groups, suggesting that the UC-MSC treatment did not increase the incidence of AEs and SAEs. **Figure 3** shows that higher heterogeneity appears in the number of types of AEs and SAEs (I^2 =^ 75%), probably due to the different types and numbers of AEs and SAEs involved in each study.

### 3.4 Secondary outcomes

#### 3.4.1 The number of patients requiring respiratory support and the duration of oxygen therapy

**Figure 4:**
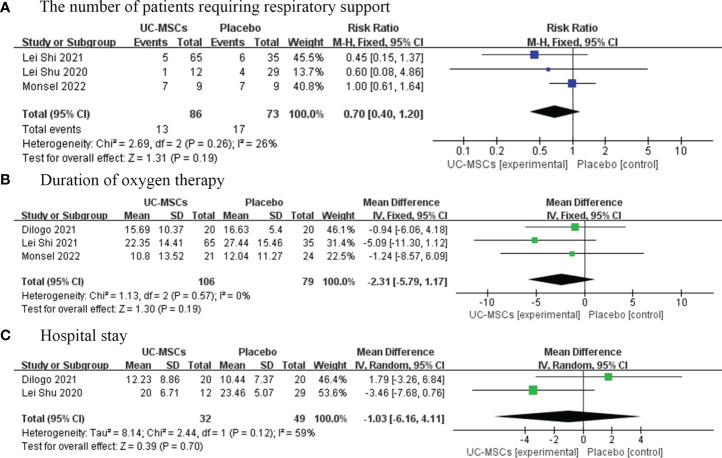
The effect of UC-MSCs therapy by forest plot diagram on the number of patients requiring respiratory support **(A)**, duration of oxygen therapy **(B)**, and hospital stay **(C)**.

We analysed three trials involving 159 patients in the experimental and control groups who required respiratory support during treatment. Meta-analysis results showed no significant difference between the UC-MSC and control groups (RR=0.70, 95% CI: [0.40, 1.20], P=0.19; **Figure 4A**). In contrast, four trials involving 203 patients analysed the time that patients needed respiratory support. We did not find significant differences in the duration of oxygenation between the UC-MSC and control groups (MD=−2.31, 95% CI: [−5.79, 1.17], P=0.19; **Figure 4B**).

##### 3.4.2 Hospital stays

Three studies have reported the average length of hospital stay of 99 patients. The results of this meta-analysis showed no significant difference in the average length of hospital stay between the UC-MSC and control groups (MD=−1.03, 95% CI: [−6.16, 4.11], P=0.70; **Figure 4C**).

### 3.5 Publication bias

**Figure 5:**
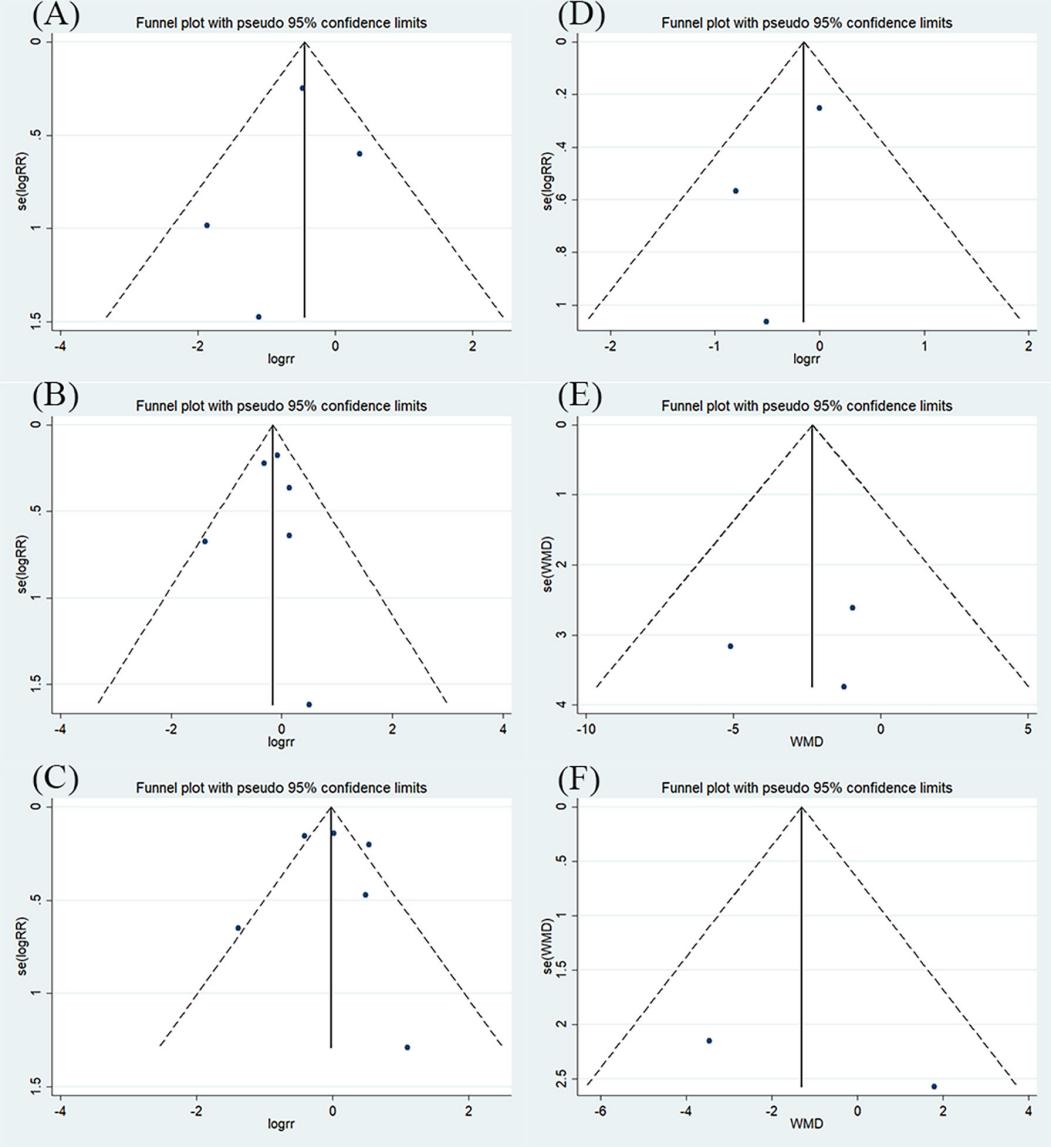
The results of funnel plot **(A)** mortality rate; **(B)** patients experiencing AEs and SAEs; **(C)** types of AEs and SAEs; **(D)** patients requiring respiratory support; **(E)** duration of oxygen therapy; **(F)** hospital stay.

Publication bias was evaluated using a funnel plot diagram. Deciding whether there is any asymmetry in the funnel plot through visual inspection **(** **Figure 5** **)**. Funnel plot symmetry of mortality rate (A), patients experiencing AEs and SAEs (B), types of AEs and SAEs (C), and duration of oxygen therapy (E), indicating a low risk of publication bias. Only two studies presented hospital length of stay (F), we could not determine its risk of publication bias. The funnel plot describing patients requiring respiratory support (D) is asymmetrical and there is potential for publication bias. It is possible that some studies with small sample sizes and statistically insignificant effects were not published. The results for the number of patients with respiratory support are affected by publication bias and this finding should be construed carefully.

## 5 Discussion

COVID-19 a novel coronavirus disease caused by the SARS-CoV-2, and its rapid spread resulted in a global pandemic. Most patients with infection have mild or moderate disease and recover within 2 to 3 weeks. However, there is still a significant risk of death in the 20% of patients who develop severe COVID-19 or even acute respiratory distress syndrome (ARDS) (41). There is evidence that MSC cell therapy has the potential to reduce all-cause mortality and improve pulmonary function (42). UC-MSC is thought to have stronger angiogenic (43) and immunomodulatory properties (44), which may be more relevant to COVID-19-induced pulmonary damage and dysregulated immune response. This systematic review and meta-analysis evaluated 10 studies of which 6 RCTs, including 293 COVID-19 patients treated with UC-MSC. Of the 149 patients evaluated in 4 RCTs, UC-MSC treatment was associated with significant reduction of 40% in all-cause mortality risk (RR=0.60, 95% CI: [0.38, 0.95], P=0.03). Three studies involving 163 patients showed no significant correlation was observed between adverse effects and UC-MSC treatment (RR=0.85, 95% CI: [0.65, 1.10], P=0.22; RR=1.00, 95% CI: [0.64, 1.58], P=1.00). However, our meta-analysis showed no significant effect of UC-MSC treatment on reducing the number of supplemental oxygen patients (RR=0.70, 95% CI: [0.40, 1.20], P=0.19), the duration of oxygen therapy (MD=−2.31, 95% CI: [-5.79, 1.17], P=0.19), or the length of hospital stay (MD=−1.03, 95% CI: [-6.16, 4.11], P=0.70).

A striking observation of our meta-analysis was that UC-MSC reduced mortality in severe COVID-19. However, immunomodulatory agents such as interleukin-6 antagonists and glucocorticoids have not been shown to significantly reduce mortality in patients with COVID-19 (45, 46). This meta-analysis of seven studies involved plasma inflammatory cells and cytokines **(** **Table 5** **)**, and significant changes in the inflammatory cytokine levels was observed in five studies (31, 32, 35, 36, 38). Three studies involved the anti-inflammatory factor IL-10 and all showed an increased level of IL-10 (31, 37, 38), with a significant increase in Monsel et al. Although the role of IL-10 in COVID-19 is unclear, IL-10 inhibits the production of pro-inflammatory cytokines, thereby reducing immune damage. However, some studies have shown that IL-10 is also increased in patients with severe COVID-19 (47), so whether UC-MSC can upregulate IL-10 levels in COVID-19 treatment has to be proven in further studies. There were seven studies involving IL-6, and five studies showed IL-6 levels were decreased after day 3 or 7 days with UC-MSC injection (31, 32, 36–38), but two studies showed no difference (30, 35). Some evidence shows that IL-6 inhibition is associated with clinical improvement in patients with COVID-19 (48). And tumor necrosis factor alpha [TNFα], an important pro-inflammatory factor, were shown to be reduced in five studies and significantly reduced in three studies (32, 35, 36). Studies have reported that the severity of COVID-19 is associated with high production of immune cells and inflammatory cytokines (49). Therefore, the beneficial effects of MSC treatment in COVID-19 patients are mediated through the regulation of inflammatory factors. Shi et al. reported that MSC therapy reduced the solid components and pulmonary fibrosis in COVID-19 patients (30). In addition, four included studies reported changes in lung imaging after MSCs treatment (30, 33, 37, 38), and the CT images showed a reduction in the area of lung inflammation, solid lung volume and gross glassy images in the UC-MSC group **(** **Table 5** **)**. Some studies show that MSCs can differentiate into type II alveolar cells and prevent lung fibrosis by inducing cell multiplication and inhibiting apoptosis (50). MSCs can act as immunomodulators and regenerate and repair damaged lung tissues in COVID-19 treatment (51). In conclusion, UC-MSC therapy reduces mortality in patients with severe COVID-19 probably by regulating immunomodulators and rescuing lung function.

**Table 5:** Secondary outcomes.

References	Immune cells/Inflammatory markers/pro-inflammatory cytokines	Pulmonary imaging changes
Lei Shi et al.	Counting of peripheral lymphocyte subsets (CD4+ T cells, CD8+ T cells, B cells, NK cells) in both groups showed no statistically significant differences between the two groups.	The proportion of solid component lung lesion volume was significantly reduced in the UC-MSCs treatment group compared to placebo.
Dilogo et al.	The expression of LIF was significantly increased in the UC-MSCs group, and LIF could suppress overactive T lymphocytes. There was an increasing trend of IL-10 and a decreasing trend of IL-6 in the experimental group compared with the control group without significant differences.	NM
Giacomo et al.	Monitoring of inflammatory cytokine concentrations (IL-5, IL-6, IL-7, TNFa, TNFb, PDGF-BB, IFNg, GM-CSF, and RANTES) from day 0 to day 6 showed a significant decrease in the UC-MSC treatment group compared to the control group.	NM
Lei Shu et al.	NM	The reduction in lung inflammation in the UC-MSCs treatment group was significantly better than that in the control group.
Monsel et al.	In the UC-MSC treatment group, inflammatory markers were significantly reduced.	NM
Kouroupis et al.	Compared with the control group, TNFα and TNFβ levels were significantly decreased and sTNFR2 levels were significantly increased in the UC-MSC group.	NM
Meng et al.	Inflammatory factors (IFN-γ, IL-1RA, MCP-1, IL-6, IP-10, IL-8, IL-18, IL-22, TNF-α, and MIP-1α) in the UC-MSCs treatment group tended to decrease over 14 days.	CT scan images of the chest showed that the lung lesions in one critically ill patient in the UC-MSCs group were well controlled within 6 days and had completely resolved within 2 weeks. In contrast, one critically ill patient in the control group had lung lesions that were still present at the time of discharge.
Wei et al.	The levels of the inflammatory cytokines TNF-a, IL-1b, and IL-6 decreased in the UC-MSCs group, while the anti-inflammatory factor IL-10 increased. the levels of IgM decreased and the levels of IgG did not change significantly.	The area of inflammation in the lungs was significantly reduced in the UC-MSCs group (p=0.003), and the number of CTs in the inflamed area also tended to recover after treatment (p=0.062).

NM, not mentioned.

A second important finding was that infusion of UC-MSC did not generate any adverse events during or after treatment. The results of our meta-analysis showed that no significant correlation was observed between adverse effects and UC-MSC treatment. In the study by Monsel et al, only one patient in the UC-MSC group developed diarrhoea, and in Meng’s study two patients developed a fever that resolved on its own after 4 hours, which were thought to be related to the treatment (35). Adverse events, such as increased lactic acid dehydrogenase levels, were not directly related to the injection of UC-MSCs. Feng et al. have observed no SAEs in patients with severe COVID-19 after 3 months of UC-MSC infusion, indicating that UC-MSC was safe for treatment in the medium term (39). In the study by Lei et al., the overall incidence of adverse events in the UC-MSC and placebo groups was similar at the 1-year follow-up. Therefore, UC-MSC injections are relatively safe for treating COVID-19.

Respiratory failure is the primary cause of death in COVID-19 patients (52). Respiratory failure in COVID-19 is a pattern of unique immune dysfunction. This unique pattern of immune dysfunction is characterised by persistent cytokine production and excessive inflammation due to low expression of IL-6-mediated human leukocyte antigen and lymphocytopaenia (53). In this systematic review, the reduction in IL-6 and inflammatory factor concentration after UC-MSC treatment. Therefore, we hypothesize that the ability of UC-MSCs to regulate inflammatory factors may play a beneficial in role slowing the development and progression of respiratory failure and thus reducing mortality. However, when respiratory support is required in severe COVID -19-related respiratory failure, the extent of lung injury outweighs the effect of UC-MSCs (54). The results of our meta-analysis also showed no significant improvement in the number of patients requiring respiratory support or in the duration of respiratory support provided by UC-MSCs. And changes in the ratio of arterial oxygen partial pressure to fractional inspired oxygen did not differ significantly between the UC−MSC and placebo groups in the study by Monsel et al. Although CT has shown that UC-MSCs improve lung injury, there is no direct evidence that UC-MSCs have an effect on improving oxygenation (54). In addition, the length of hospital stay was not reduced in patients treated with UC-MSCs, suggesting that UC-MSCs treatment did not shorten the recovery time of COVID-19 patients. But there are too few studies and further studies are needed to be concluded.

Two prospective cohort studies were conducted with patients from previous RCTs with 3-month and 1-year follow-ups **(** **Table 6** **)**. Pulmonary imaging showed that the UC-MSC group had more normal CTs than that presented by the control group after 6 and 12 months (40). The values of the pulmonary function test, forced expiratory volume in 1 s (mean FEV1) and FEV1/forced vital capacity (FVC) were higher in the UC-MSC group than those in the control group. In the long-term follow-up, the 6-minute walk distance (6-MWD) showed a numerical increase at each follow-up point for patients treated with UC-MSCs compared to that of the placebo group (40). Although both studies impart a positive prognostic effect for UC-MSC treatment, there are too few studies to draw this conclusion and we need more studies to support this view.

**Table 6:** Prognosis.

References	Pulmonary imaging changes	Pulmonary function testing	Constitutionalsymptoms	AEs/SAEs
Lei et al.	A large number of patients are discharged from the hospital with sequelae like fibrous stripes, GGO, air bronchogram sign, interlobular septal thickening, crazy-paving pattern, and honeycomb pattern. After 6 months, 6 patients in the MSC group had normal CT images, but patients in the placebo group did not exhibit normal CT findings. after 12 months, 10 patients in the MSC group had normal CT images, but none were found in the placebo group at month 12.	The 6-MWD showed a numerical increase in the distance at each follow-up point for patients treated with UC-MSCs compared to the placebo group.	The incidence of sleep difficulties, fatigue, muscle weakness, and pain were lower in the UC-MSCs group than in the control group.	The incidence of adverse events was similar in the UC-MSCs and placebo groups at the 1-year follow-up. The common adverse event in both groups was an increase in lactate dehydrogenase.
Feng et al.	CT imaging was performed to assess lung changes. After three months, no significant adverse effects were observed in the UC-MSC group.	The SGRQ score was significantly lower in the UC-MSCs group compared with the control group (P<0.05). The mean FEV1 and FEV1/FVC ratios were higher in the UC-MSC group compared with the control group (P<0.05).	The incidence of wheezing was significantly lower in the UC-MSCs group than in the control group.	There were no serious adverse events in the UC-MSCs treatment group during the 3 months of follow-up.

GGO, Ground-glass opacity.

Currently, no studies have shown that UC-MSCs are better than other sources of MSCs for COVID-19 treatment. However, compared with other MSC sources (bone marrow, adipose tissue, etc.), UC-MSCs have a high proliferation capacity, rapid self-renewal, more stable doubling time, low immunogenicity and a more straightforward harvesting process (19). Therefore, human umbilical cord tissue may be an optimal source of adult multipotent stem cells.

This study has the following limitations. First, although this analysis supported that UC-MSC therapy reduces mortality in patients with severe COVID-19, it was important to note that SARS-CoV-2 Variants of Interest (VOIs) were not involved in our included studies, so it was not known whether UC-MSC had a therapeutic effect on VOIs as well. In addition, The conclusions were drawn from a comparison of UC-MSC with earlier standard care and did not take into account new treatments and vaccinations (55). Recently, numerous studies had confirmed that vaccination reduces mortality in patients with COVID-19 (56), and further validation is needed to determine whether the vaccine interacts with UC-MCS therapy.

Second, although our analysis found no increase in the incidence of AEs and SAEs with UC-MSC treatment, this finding should be interpreted with caution given that the types and numbers of adverse events reported were not consistent across studies.

Third, only a few stem cells from other sources were used in clinical trials of COVID-19, and it is not possible to accurately compare UC-MSC with MSC from other sources.

Fourth, only 10 studies were included in our meta-analysis; the test power of our analysis might have increased with more RCTs.

Fifth, heterogeneity included in the study was inevitable due to differences in race, age, disease severity, comorbidities, combination drug therapy, evaluation criteria and dose administered.

Sixth, although the funnel plots of some studies showed low publication bias, any potential publication bias cannot be ruled out due to the small number of studies or sample size, and visual bias of the evaluator.

## 5 Conclusions

In this systematic review and meta-analysis, UC-MSCs were found to be safe and effective for the treatment of COVID-19. Compared to other sources of MSCs, UC-MSCs are easier and safer to obtain and produce and can be beneficial for the clinical promotion of treatment. However, whether UC-MSCs are superior to other sources of MSCs in the treatment of COVID-19 requires more clinical trial evidence.

## Data availability statement

The original contributions presented in the study are included in the article/**Supplementary Material**. Further inquiries can be directed to the corresponding authors.

## Author contributions

C-WY proposed the study idea, searched the database, screened and evaluated the evidence and wrote the manuscript. R-DC collected and organized the data and extracted the data from the tables and S-JH revised the manuscript and edited the English. M-JK made changes to this article. Q-RZ made the figures and tables. All authors have read and approved the final manuscript.

## Funding

This study received funding from the National Natural Science Foundation of China (NO. 82002302) and the Natural Science Foundation of Shandong Province (NO. ZR2020QH074).

## Acknowledgments

The authors thank Dr Dachuan Wang (Shandong Provincial Hospital affiliated with Shandong University) for his valuable feedback and critical review of the draft manuscript.

## Conflict of interest

The authors declare that the research was conducted in the absence of any commercial or financial relationships that could be construed as a potential conflict of interest.

## Publisher’s note

All claims expressed in this article are solely those of the authors and do not necessarily represent those of their affiliated organizations, or those of the publisher, the editors and the reviewers. Any product that may be evaluated in this article, or claim that may be made by its manufacturer, is not guaranteed or endorsed by the publisher.

## Supplementary material

Additional file 1 PRISMA 2020 checklist.

## Abbreviations

UC-MSCs, umbilical cord mesenchymal stromal cells; SARS-CoV-2, syndrome coronavirus 2; CRS, cytokine release syndrome; MSCs, mesenchymal stem cells; RCTs, randomized controlled trials; CSs, cohort Studies; AEs, adverse events; SAEs, severe adverse events; ROBINS-I, Risk of Bias In Non-randomized Studies of Interventions; RR, relative risks; OR, odds ratio; SRF, severe respiratory failure; VEGF, vascular endothelial growth factor; FEV1, forced expiratory volume in one second; FVC, forced vital capacity; 6-MWD, 6 minutes walk distance; SGRQ, St.George’s Respiratory Questionnaire.
