# Corrigendum: Efficacy of umbilical cord mesenchymal stromal cells for COVID-19: a systematic review and meta-analysis

**DOI:** 10.3389/fimmu.2023.1344990

**Published:** 2024-01-04

**Authors:** Cong-wen Yang, Ru-dong Chen, Qing-run Zhu, Shi-jie Han, Ming-jie Kuang

**Affiliations:** ^1^ Department of Neurosurgery, Weifang Medical University, Weifang, China; ^2^ Department of Orthopedics, Shandong Provincial Hospital Affiliated to Shandong First Medical University, Jinan, Shandong, China

**Keywords:** COVID-19, umbilical cord mesenchymal stromal cells (UC-MSCs), immunomodulation, adverse events and severe adverse events, the Mortality rate

## Incorrect affiliation

In the published article, there was an error in the second affiliation. Instead of “Department of Spinal Surgery, Provincial Hospital Affiliated to Shandong First Medical University, Jinan, China”, it should be “Department of Orthopedics, Shandong Provincial Hospital Affiliated to Shandong First Medical University, Jinan, Shandong, China”.

The authors apologize for this error and state that this does not change the scientific conclusions of the article in any way. The original article has been updated.

